# Supplementary material for: Role of New Chiral Additives on Physical-Chemical Properties of the Nematic Liquid Crystal Matrix
Source: Materials (Basel). 2023 Sep 2;16(17):6038. doi: 10.3390/ma16176038 (PMC10488559; doi:10.3390/ma16176038)
Supplement: Supplementary file 1 [file materials-16-06038-s001.zip › materials-2542877-supplementary.pdf]

ROLE OF NEW CHIRAL ADDITIVES ON PHYSICAL-CHEMICAL PROPERTIES OF THE NEMATIC LIQUID CRYSTAL MATRIX

Alexey S. Merekalov[1], Oleg N. Karpov[1], Georgiy A. Shandryuk[1], Olga A. Otmakhova[1], Alexander V. Finko[1,2], Artem V. Bakirov[3], Vladimir S. Bezborodov[4] and Raisa V. Talroze[1,*]

[1] A.V. Topchiev Institute of Petrochemical Synthesis of the Russian Academy of Sciences, Moscow 119991, 29 Leninsky Pr., Russia
[2] Department of Chemistry, Moscow State University, Moscow 119991, Leninskie Gory 1/3, Russia.
[3] N.S. Enikolopov Institute of Synthetic Polymeric Materials, Russian Academy of Sciences, Moscow 117393, 70 Profsoyuznaya Str., Russia
[4] Belarusian State Technological University, 220006, Minsk, 13a Sverdlova Str., Belarus
*Correspondence address: rtalroze@ips.ac.ru

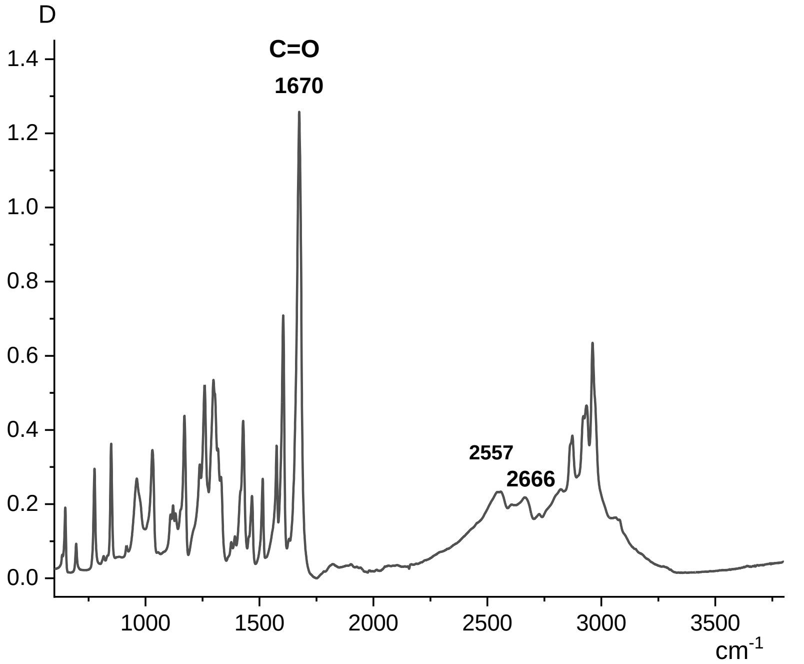

a

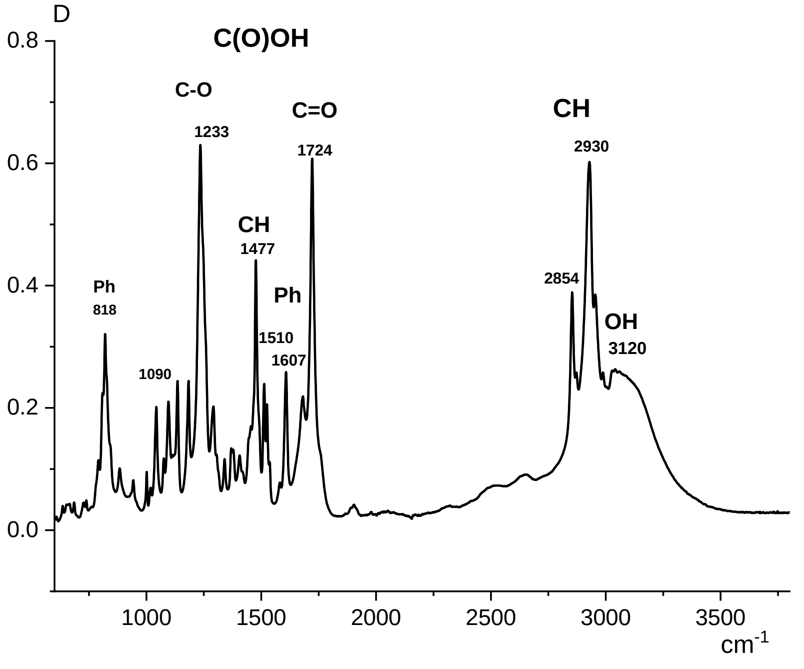

b

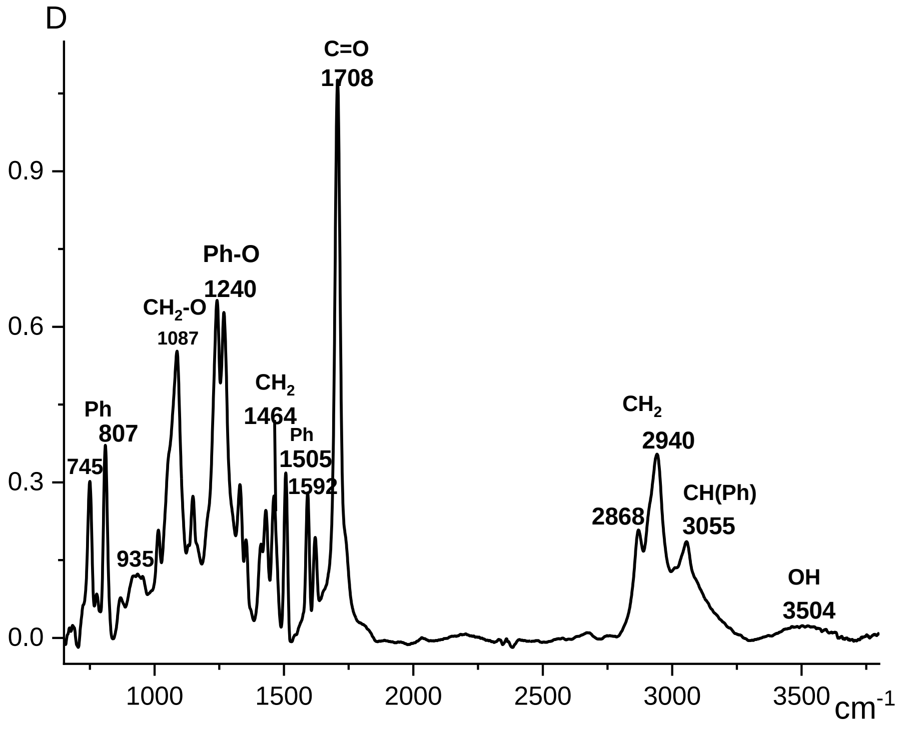

c

Figure S1. FTIR spectra of CA1 (a), CA2 (b), CA3 (c).

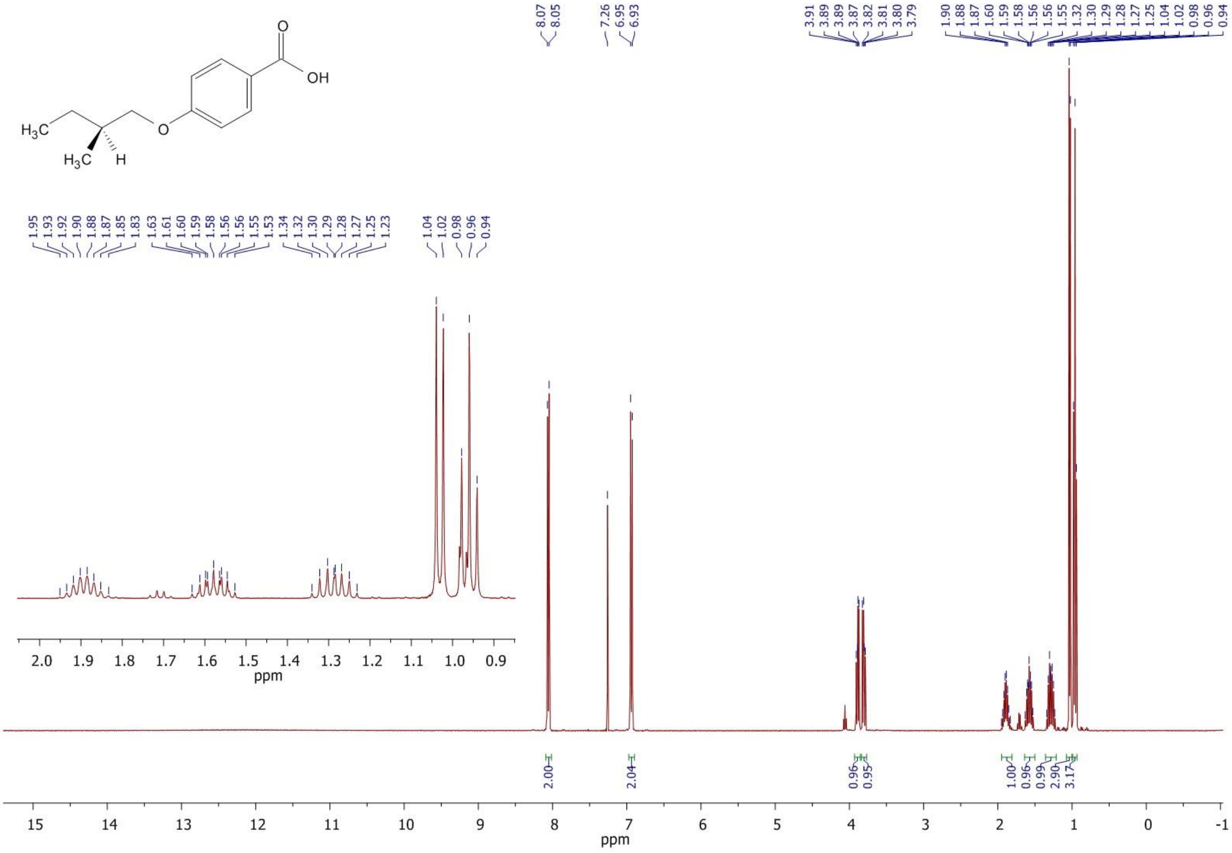

a

^{1}H NMR (400 MHz, CDCl3) δ (ppm) 0.96 (t, J = 7.5 Hz, 3H), 1.03 (d, J = 6.8 Hz, 3H), 1.23 – 1.34 (m, 1H), 1.53 – 1.63 (m, 1H), 1.83 – 1.95 (m, 1H), 3.81 (dd, J = 9.0, 6.6 Hz, 1H), 3.89 (dd, J = 9.0, 6.0 Hz, 1H), 6.94 (d, J = 8.9 Hz, 2H), 8.06 (d, J = 8.8 Hz, 2H).

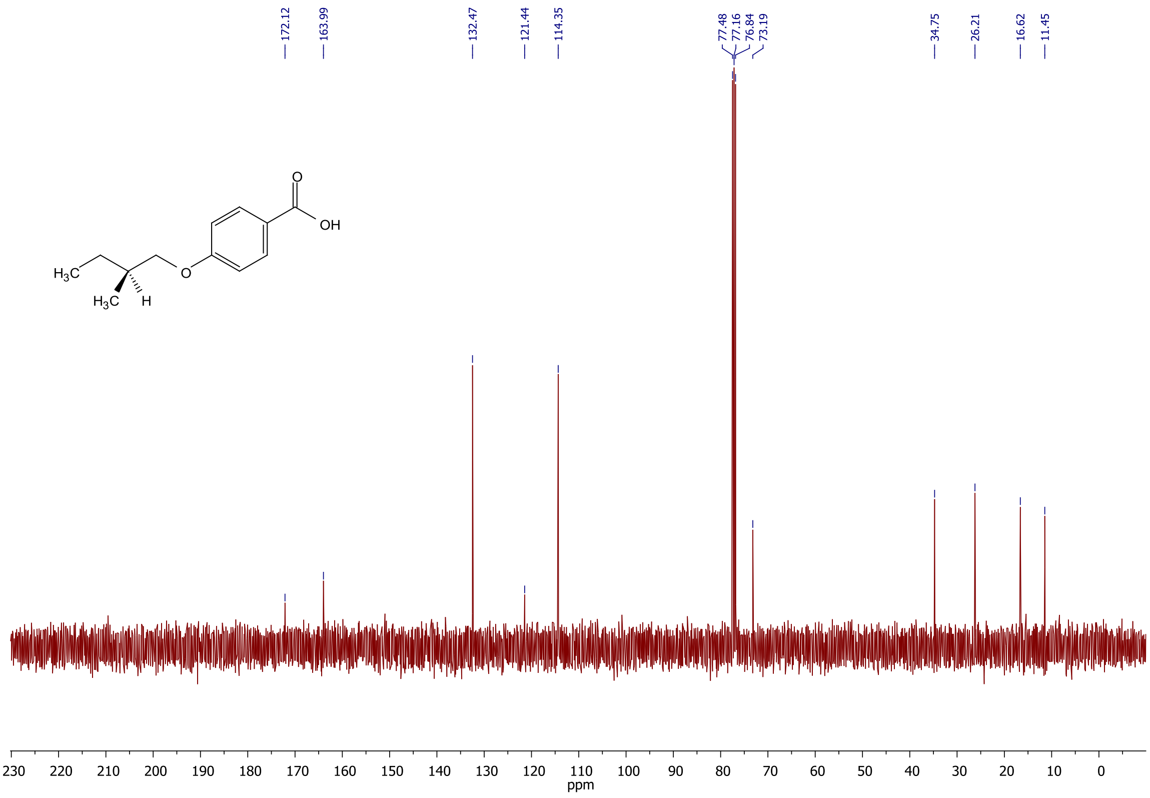

b

^{13}C {^{1}H} NMR (101 MHz, $CDCl_3$) δ (ppm) 11.4, 16.6, 26.2, 34.7, 73.2, 114.3 (2C), 121.4, 132.5 (2C), 164.0, 172.1.

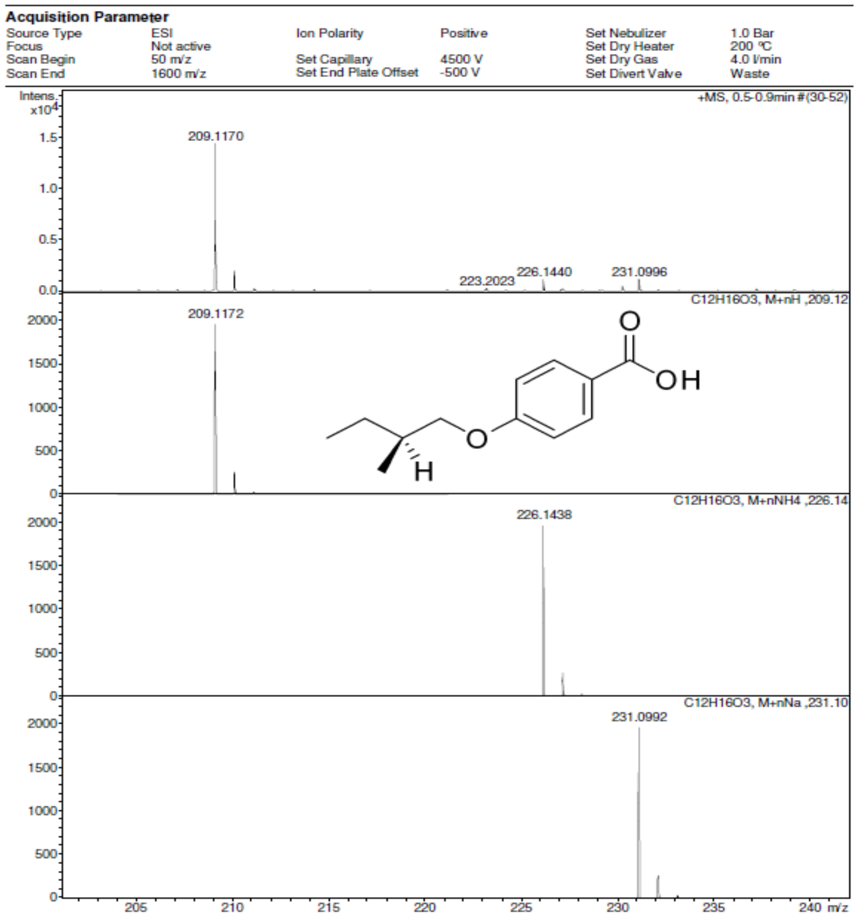

c

HRMS (FTMS + cESI) m/z: $[M+H]^+$, calculated for $C_{12}H_{16}O_3$ 209,1172; found 209.1170.

Figure S2. ^{1}HNMR (a), ^{13}NMR (b) and mass-spectra (c) of CA1

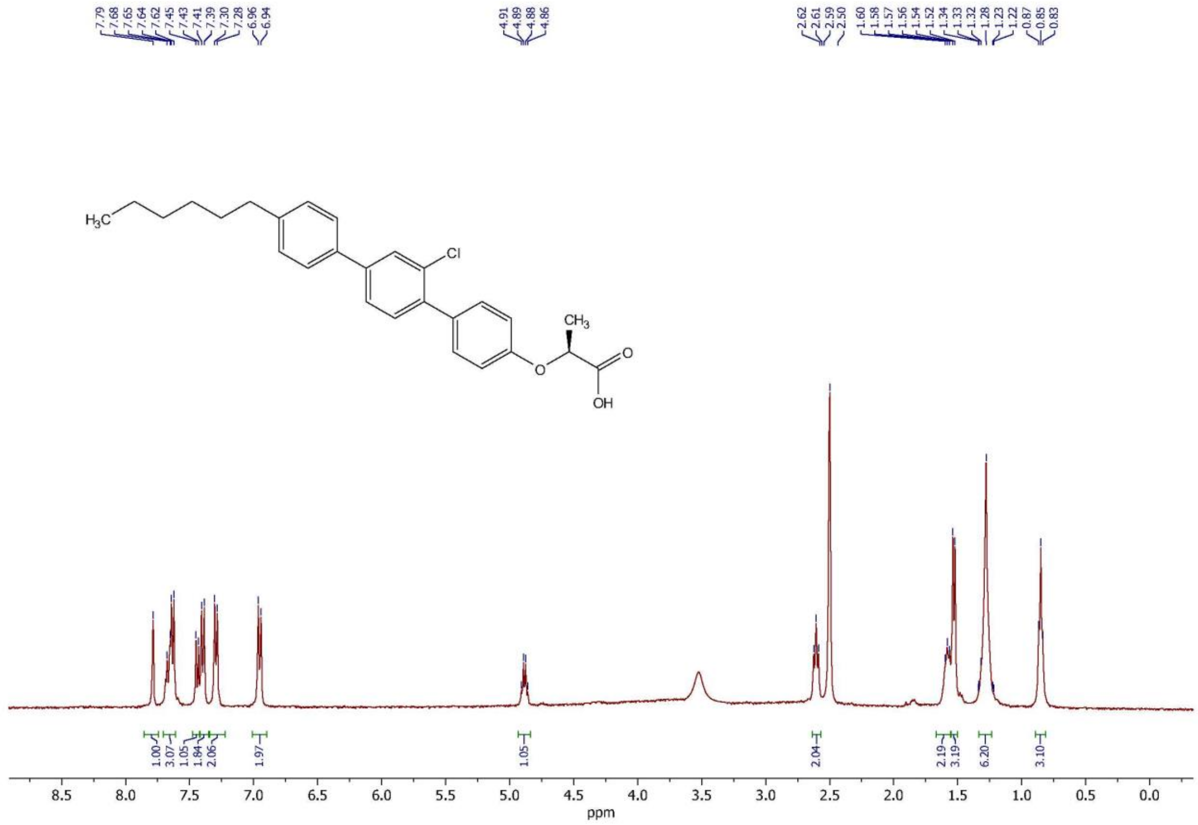

^{1}H NMR (400 MHz, DMSO-d6) δ (ppm) 0.85 (t, J = 6.3 Hz, 3H, CH_3), 1.22 – 1.34 (m, 6H, 3×CH_2), 1.53 (d, J = 6.6 Hz, 3H, Me), 1.56 – 1.60 (m, 2H, CH_2), 2.61 (t, J = 7.5 Hz, 2H, CH_2), 4.88 (q, J = 6.6 Hz, 1H, CH), 6.95 (d, J = 8.5 Hz, 2H, H_{Ar}), 7.29 (d, J = 7.9 Hz, 2H, H_{Ar}), 7.40 (d, J = 8.4 Hz, 2H, H_{Ar}), 7.44 (d, J = 8.0 Hz, 1H, H_{Ar}), 7.62 – 7.69 (m, 3H, H_{Ar}), 7.78 (d, J = 1.4 Hz, 1H, H_{Ar}).

Figure S3. ^{1}H NMR of CA2

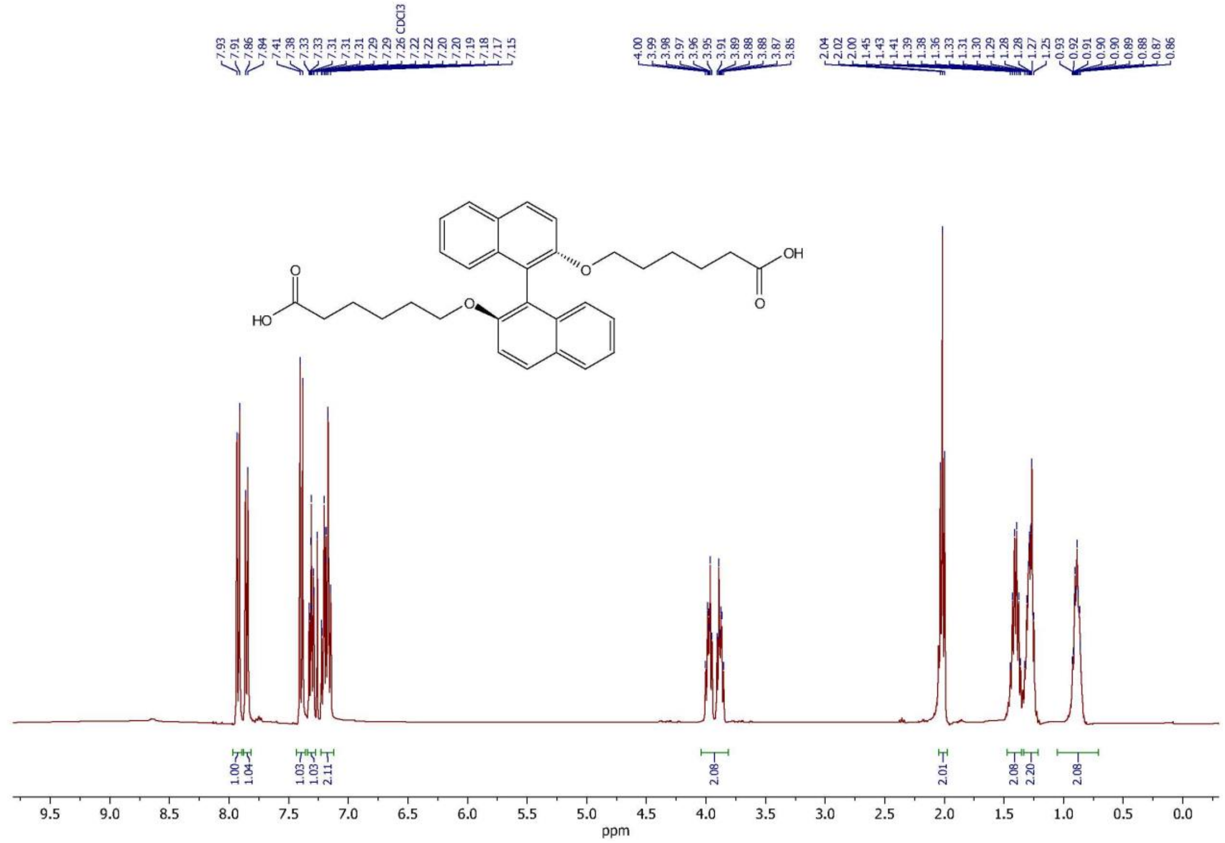

^{1}H NMR (400 MHz, $CDCl_3$) δ 7.92 (d, *J* = 9.0 Hz, 1H), 7.85 (d, *J* = 8.2 Hz, 1H), 7.39 (d, J = 9.1 Hz, 1H), 7.31 (ddd, J = 8.2, 6.5, 1.5 Hz, 1H), 7.15 – 7.22 (m, 2H), 3.85 – 4.00 (m, 2H), 2.02 (t, J = 7.5 Hz, 2H), 1.36 – 1.45 (m, 2H), 1.25 – 1.33 (m, 2H), 0.86 – 0.93 (m, 2H).

Figure S4. ^{1}H NMR spectrum of CA3

Table S1. Elemental Analysis of CA1 (a), CA2 (b), CA3 (c).

CA1 (a)	Calculated (%): C 69.21; H 7.74; O 23.05; Found (%): C 68.95; H 7.92; O 23.13.
CA2 (b)	Calculated (%): C 74.21; H 6.69; Cl 8.11; O 10.98; Found (%): C 74.35; H 6.76; O 11.10.
CA3 (c)	Calculated (%): C, 74.69; H, 6.66; O, 18.65; Found (%): C 74.60; H 6.78; O 18.62.

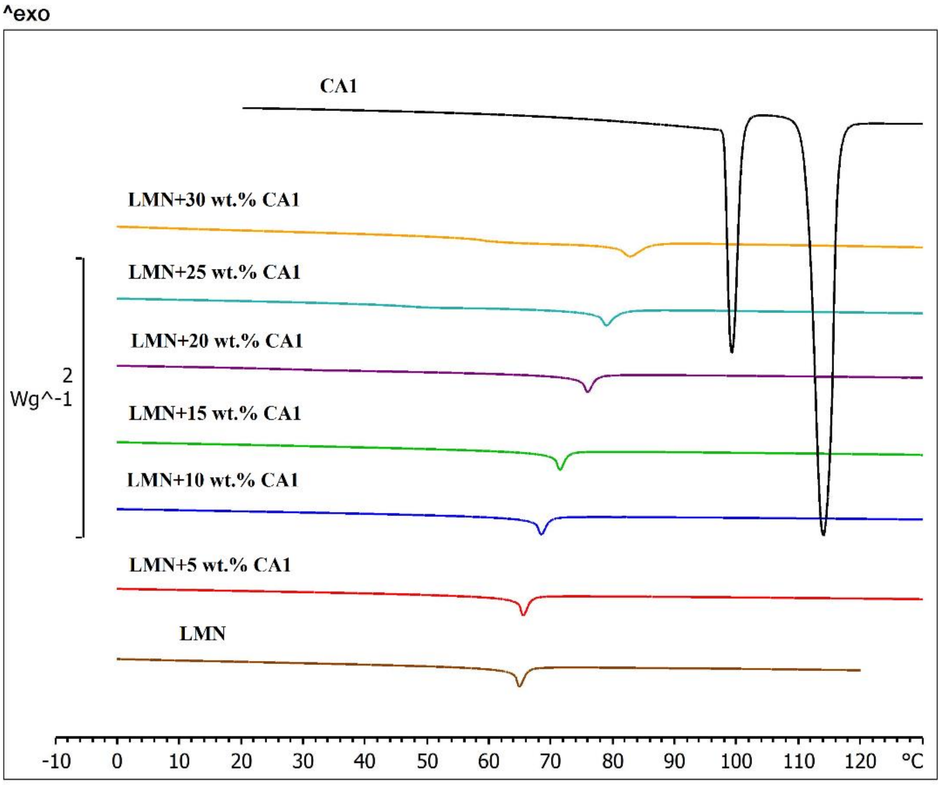
^exo
CA1
LMN+30 wt.% CA1
LMN+25 wt.% CA1
LMN+20 wt.% CA1
LMN+15 wt.% CA1
LMN+10 wt.% CA1
LMN+5 wt.% CA1
LMN
2
Wg^-1
-10 0 10 20 30 40 50 60 70 80 90 100 110 120 °C

a

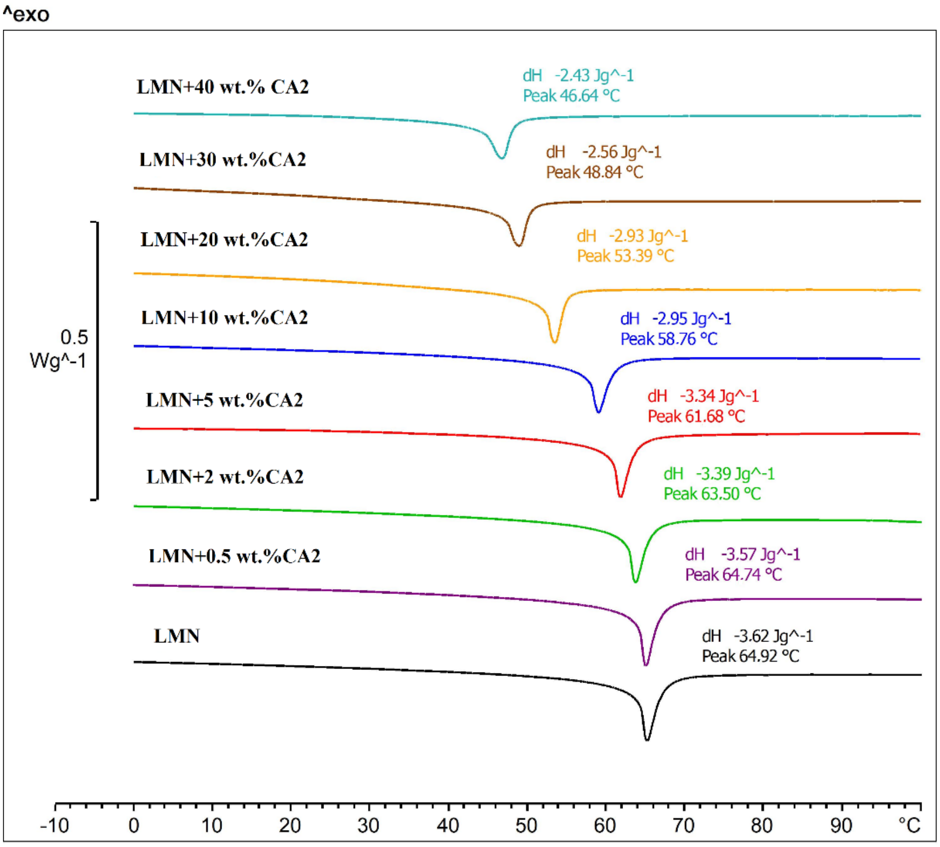
^exo
LMN+40 wt.% CA2
dH -2.43 Jg^-1
Peak 46.64 °C
LMN+30 wt.%CA2
dH -2.56 Jg^-1
Peak 48.84 °C
LMN+20 wt.%CA2
dH -2.93 Jg^-1
Peak 53.39 °C
LMN+10 wt.%CA2
dH -2.95 Jg^-1
Peak 58.76 °C
LMN+5 wt.%CA2
dH -3.34 Jg^-1
Peak 61.68 °C
LMN+2 wt.%CA2
dH -3.39 Jg^-1
Peak 63.50 °C
LMN+0.5 wt.%CA2
dH -3.57 Jg^-1
Peak 64.74 °C
LMN
dH -3.62 Jg^-1
Peak 64.92 °C
0.5
Wg^-1
-10 0 10 20 30 40 50 60 70 80 90 °C

b

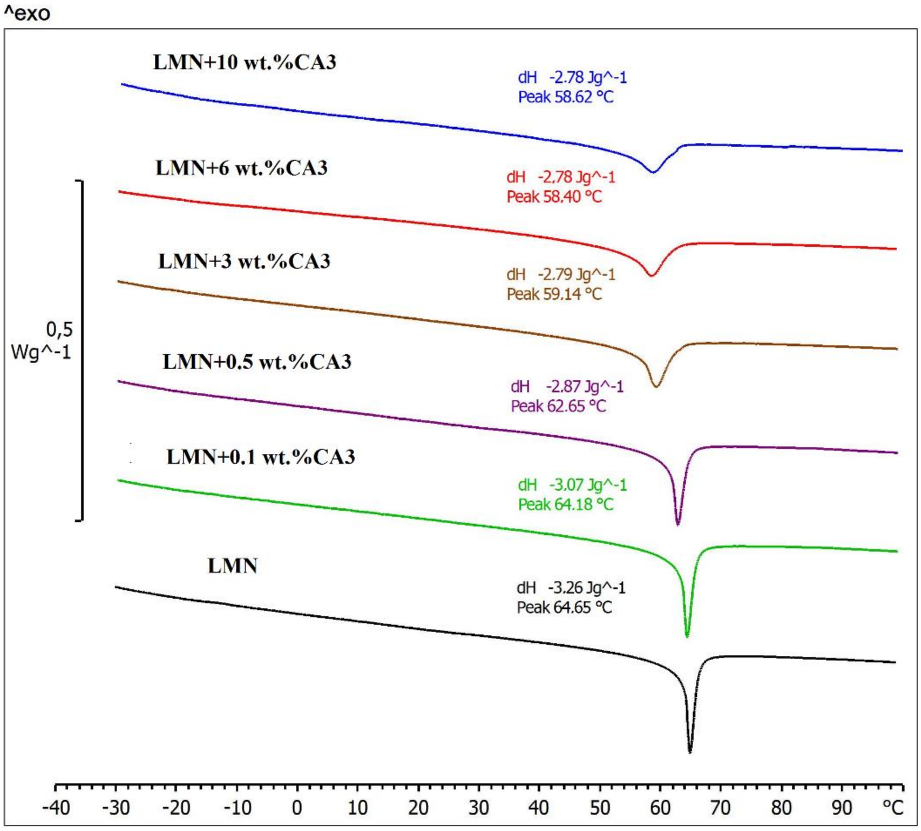

c

Figure S5. DSC curves of LMN in blends with CA1 (a), CA2(b) and CA3 (c).

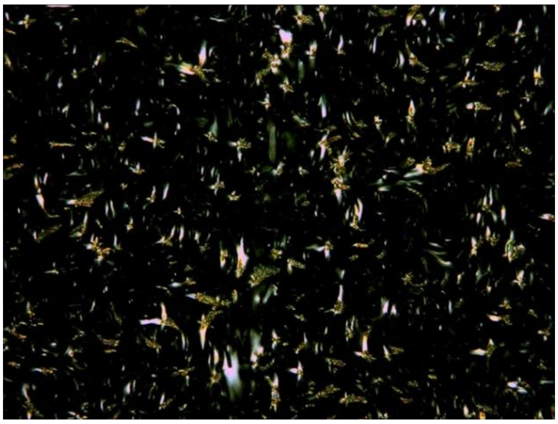

Figure S6 The optical textures (in crossed polarizers) of LMN with 25% CA1 at 22 °C

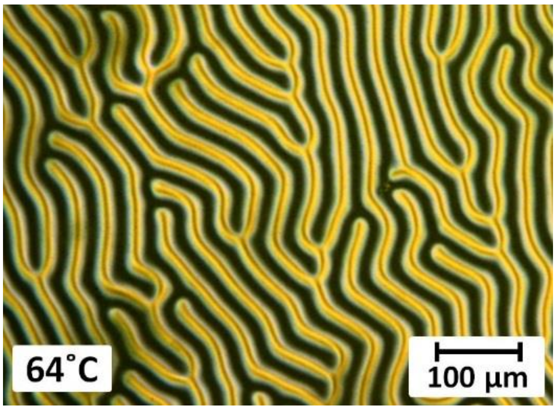

a 15% CA1 heating

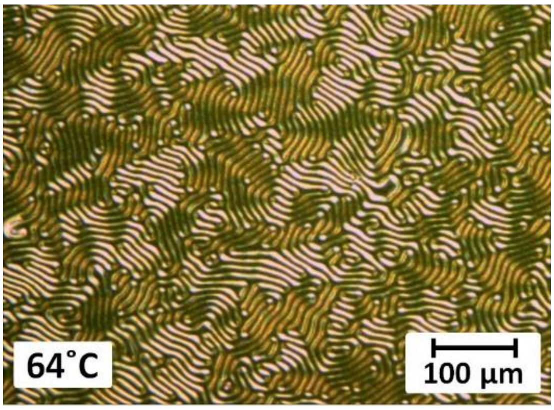

b 15% CA1 cooling

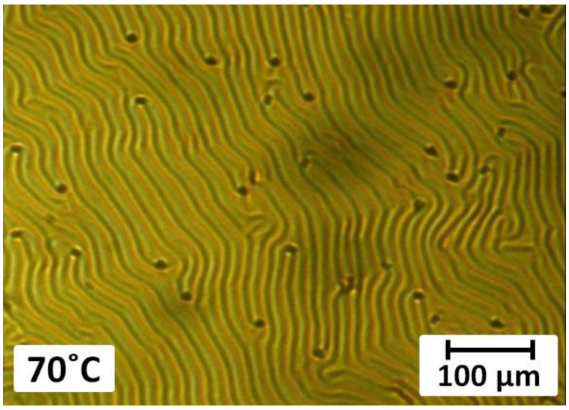

c 20% CA1 heating

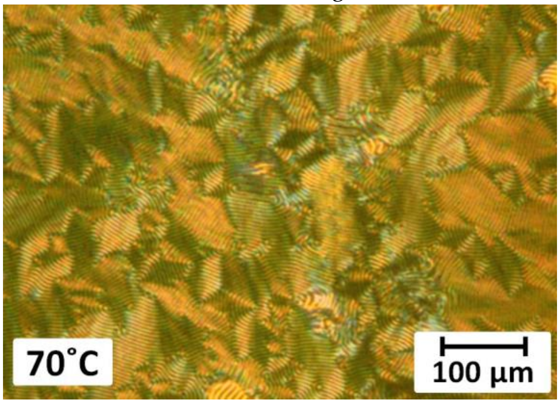

d 20% CA1 cooling

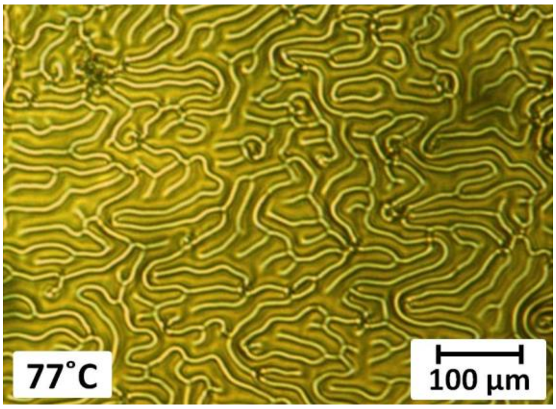

e 25% CA1 heating

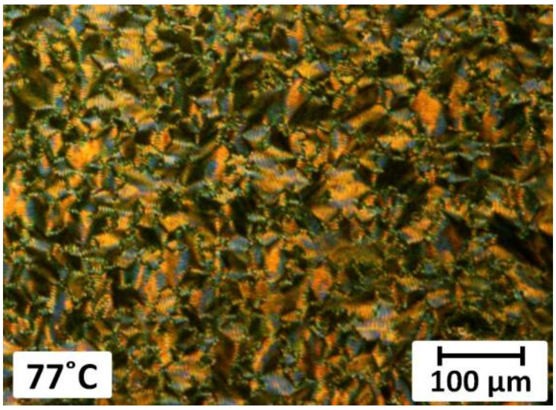

f 25% CA1 cooling

Figure S7 The optical textures (in crossed polarizers) of LMN-CA1 mixtures with different concentration: a,b – 15%, c,d – 20%, e,f – 25%

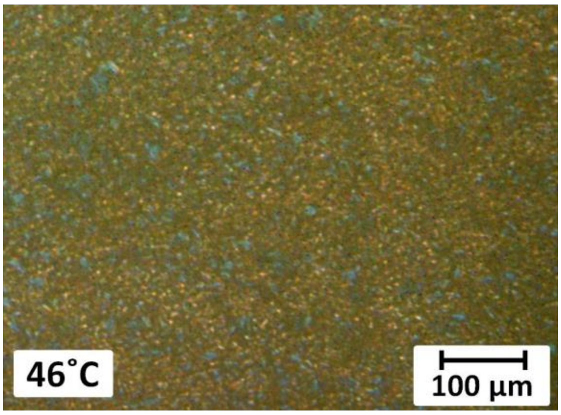

a

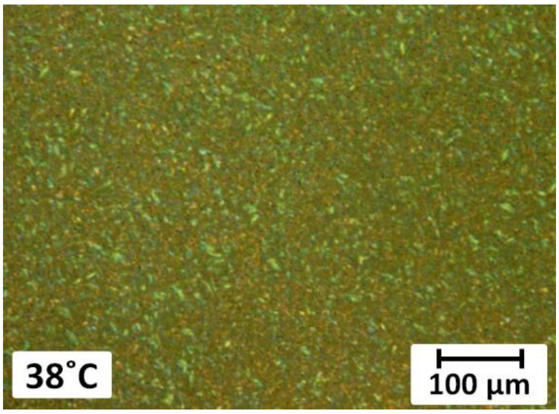

b

Figure S8. The optical textures (in crossed polarizers) of LMN- 20 wt.% CA2 mixtures: a –heating, b–cooling
